# Microsecond Time-Resolved Absorption Spectroscopy Used to Study CO Compounds of Cytochrome *bd* from *Escherichia coli*

**DOI:** 10.1371/journal.pone.0095617

**Published:** 2014-04-22

**Authors:** Sergey A. Siletsky, Andrey A. Zaspa, Robert K. Poole, Vitaliy B. Borisov

**Affiliations:** 1 Belozersky Institute of Physico-Chemical Biology, Lomonosov Moscow State University, Moscow, Russian Federation; 2 Department of Molecular Biology and Biotechnology, The University of Sheffield, Sheffield, United Kingdom; National Research Council of Italy (CNR), Italy

## Abstract

Cytochrome *bd* is a tri-heme (*b*_558_, *b*_595_, *d*) respiratory oxygen reductase that is found in many bacteria including pathogenic species. It couples the electron transfer from quinol to O_2_ with generation of an electrochemical proton gradient. We examined photolysis and subsequent recombination of CO with isolated cytochrome *bd* from *Escherichia coli* in one-electron reduced (MV) and fully reduced (R) states by microsecond time-resolved absorption spectroscopy at 532-nm excitation. Both Soret and visible band regions were examined. CO photodissociation from MV enzyme possibly causes fast (τ<1.5 µs) electron transfer from heme *d* to heme *b*_595_ in a small fraction of the protein, not reported earlier. Then the electron migrates to heme *b*_558_ (τ∼16 µs). It returns from the *b*-hemes to heme *d* with τ∼180 µs. Unlike cytochrome *bd* in the R state, in MV enzyme the apparent contribution of absorbance changes associated with CO dissociation from heme *d* is small, if any. Photodissociation of CO from heme *d* in MV enzyme is suggested to be accompanied by the binding of an internal ligand (L) at the opposite side of the heme. CO recombines with heme *d* (τ∼16 µs) yielding a transient hexacoordinate state (CO-Fe^2+^-L). Then the ligand slowly (τ∼30 ms) dissociates from heme *d*. Recombination of CO with a reduced heme *b* in a fraction of the MV sample may also contribute to the 30-ms phase. In R enzyme, CO recombines to heme *d* (τ∼20 µs), some heme *b*_558_ (τ∼0.2–3 ms), and finally migrates from heme *d* to heme *b*_595_ (τ∼24 ms) in ∼5% of the enzyme population. Data are consistent with the recent nanosecond study of Rappaport et al. conducted on the membranes at 640-nm excitation but limited to the Soret band. The additional phases were revealed due to differences in excitation and other experimental conditions.

## Introduction

A *bd*-type terminal respiratory oxidase has been found only in prokaryotes [1]. Cytochrome *bd* couples the electron transfer from quinol to molecular oxygen (reducing the latter to water) with generation of an electrochemical proton gradient across the cytoplasmic membrane [2]–[5]; however the energetic efficiency of such coupling is two times lower than that of the cytochrome *bo*_3_ [6], and *aa*_3_-type cytochrome oxidases [8], [9]. The cytochrome *bd* oxidase usually prevails in bacterial respiratory chains under low oxygen conditions [10] and is isolated as a stable oxygenated complex [11]–[14] in agreement with its high affinity for O_2_ [15]–[17]. In addition to its bioenergetic function, cytochrome *bd* serves a number of important physiological roles [1], [10], [18]–[20] including protection of bacteria against stress caused by nitric oxide [21]–[25] or hydrogen peroxide [26]–[29].

In contrast to heme-copper oxidases whose various members have been investigated in great detail (reviewed by [8], [9], [30]) cytochrome *bd* remains poorly studied. Its three-dimensional structure has not been solved yet. This integral membrane protein is known to be composed of two different subunits carrying three hemes, *b*_558_, *b*_595_, and *d*, which are likely located near the periplasmic side of the membrane [10]. The low-spin hexacoordinate heme *b*_558_ seems to be directly involved in the quinol oxidation, and the two quinol protons are released into the periplasm. His_186_ and Met_393_ of subunit I were identified as its axial ligands [31], [32]. The high-spin heme *d* is the core of the enzyme where O_2_ is bound, activated and reduced into H_2_O. The protons required to reduce O_2_ are most likely taken from the cytoplasmic side of the membrane via an extended transmembrane H^+^-pathway [3], [5], similarly to the typical *aa*_3_-type oxidases. Thus, the charge separation results in an electrochemical proton gradient across the membrane. According to current thinking [14], cytochrome *bd in vivo* undergoes the following catalytic transformations: R → OXY → P → F → R, where R, OXY and F are respectively reduced, oxygenated and ferryl forms of heme *d*. A short-lived intermediate P discovered by [4] is possibly a heme *d* ferryl porphyrin π-cation radical [33]. The nature of the heme *d* axial ligand is not known with certainty, although this might be Glu_99_ of subunit I [34].

The oxygen reductase site of most known terminal oxidases is binuclear; it is either heme-copper (proton-translocating heme-copper oxidases) [8], [9], [30], heme-heme, or contains two non-heme-iron atoms (non-coupled alternative oxidases as in plants and certain bacteria) [10]. Therefore, a key issue in studies of the functioning of *bd*-type terminal oxidases is to understand how their O_2_-reducing site is arranged; how many metal redox-active groups, one or two, are involved. In this regard, the role of heme *b*_595_ is of special interest. It is the high-spin pentacoordinate heme [35] ligated by His_19_ of subunit I [36] and can mediate electron transfer from heme *b*_558_ to heme *d* [37]. A number of data suggest that this heme forms a di-heme active site with heme *d* [3], [35], [38]–[48]. Other authors believe that cytochrome *bd* does not possess a bimetallic oxygen reductase site [49]. Finally, it was suggested that heme *b*_595_ serves a second, additional O_2_ binding site in the enzyme [15].

Pulsed laser spectroscopy with microsecond time resolution allows tracking of real-time changes in an individual heme site induced by photodissociation of a ligand and concomitant electron transfer processes. Examination of photolysis of the CO complex and subsequent recombination of the ligand to the enzyme may allow modeling the reaction of the oxidase with its natural substrate, O_2_. Interaction of CO with cytochrome *bd* found in many pathogens [10], [19] is also of interest in light of the recent data on a possible use of this respiratory poison as a new antibacterial agent [50], [51]. In this work, we have compared the flash-induced recombination of CO to partially and fully reduced isolated cytochrome *bd* from *Escherichia coli* on the micro/millisecond time scale using 532-nm excitation (*E. coli* has two *bd*-type terminal oxidases, named *bd*-I and *bd*-II; unless otherwise stated, we refer to cytochrome *bd*-I throughout the paper).

## Materials and Methods

### Bacterial strain

The strain of *Escherichia coli* GO105 devoid of cytochrome *bo*_3_ and cytochrome *bd* oxidases and harboring plasmid pTK1 with the genes encoding cytochrome *bd* was used for overexpressing cytochrome *bd* [32].

### Cell growth, membrane preparation, isolation and purification of enzyme

Bacterial cells were grown aerobically in a 10 L stirred fermentor or in flasks on a shaker (at 200 rpm) in the medium described in [44]. To obtain subcellular vesicles, the cells were disrupted by passing the cell suspension through a French press as reported [44]. The *bd*-type quinol oxidase was isolated and purified as described previously [44], [52].

### Sample preparation, enzyme concentration and assay conditions

The CO complexes of the isolated enzyme in the MV (one-electron-reduced “mixed-valence”, *b*_558_^3+^ *b*_595_^3+^ *d* ^2+^) and R (dithionite-reduced (fully reduced), *b*_558_^2+^ *b*_595_^2+^ *d* ^2+^) states were generated as described in [41], [46]. To generate MV-CO, the air-oxidized cytochrome *bd* (which is manly in the one-electron-reduced oxygenated form, MV-O_2_) was purged with argon and then with 1 mM CO. In some experiments MV-CO was prepared by oxidation of cytochrome *bd* in the R-CO state with small quantities of air. R-CO was obtained by adding a few grains of solid sodium dithionite to cytochrome *bd* in the MV-CO state or by bubbling of the fully reduced enzyme (R) with 1 mM CO. Cytochrome *bd* concentration was determined from the difference absorbance spectra (dithionite-reduced *minus* “air-oxidized”) using Δ*ε*_628–607_ of 10.8 mM^−1^ cm^−1^ [35]. CO concentration was estimated assuming its solubility in water at 20 °C and 1 atm to be 1 mM. The measurements were performed at 20 °C in 50 mM Hepes, 50 mM Ches, 0.1 mM EDTA, and 0.05% sodium *N*-lauryl-sarcosinate (pH 8.0) in an optical cell of 10 mm pathway. Concentrations of cytochrome *bd* and CO were 1.9 µM and 1 mM respectively.

### Spectroscopy

Static absorbance spectra were recorded using a SLM Aminco DW-2000 UV/Vis spectrophotometer (SLM Instruments). To examine flash-induced dissociation of CO from cytochrome *bd* and kinetics of the subsequent recombination on the micro/millisecond time scale, a Nd:YAG laser (Quantel model 481, the second harmonics with the 532-nm excitation wavelength, a pulse duration of 15 ns and pulse energies of 40–120 mJ) was used. Monitoring light from a 75-Watt halogen lamp was filtered through a Jobin Yvon grating monochromator with slit width of 2–8 mm and passed through the sample positioned in a thermostated compartment. After passing through the sample, the monitoring light was passed to the photomultiplier via a second grating monochromator and glass filters with specific light transmission characteristics (OD_540_/OD_450_ > 7.5 or OD_580_/OD_680_ > 140). The signal from the photomultiplier is recorded with a PC-interfaced digital transient recorder (Datalab 1080). To improve the signal-to-noise ratio, 50–250 kinetic traces were acquired at 5 s intervals. With a reflecting mirror the excitation beam enters the sample compartment within the area of monitoring light that passes through the sample perpendicular to the excitation beam. The measurement setup was described in detail in [53]–[55]. Flash-induced absorbance changes of the enzyme were recorded at selected wavelengths shown in the Figures.

### Analysis

Data treatment was carried out using the software packages GIM (Graphic Interactive Management) developed by Dr. Alexander L. Drachev (subroutine “Discrete”), PLUK developed by Dr. Yannis Kalaidzidis, Origin (OriginLab Corporation) and MATLAB (The Mathworks, South Natick, MA).

## Results

**Figure 1:**
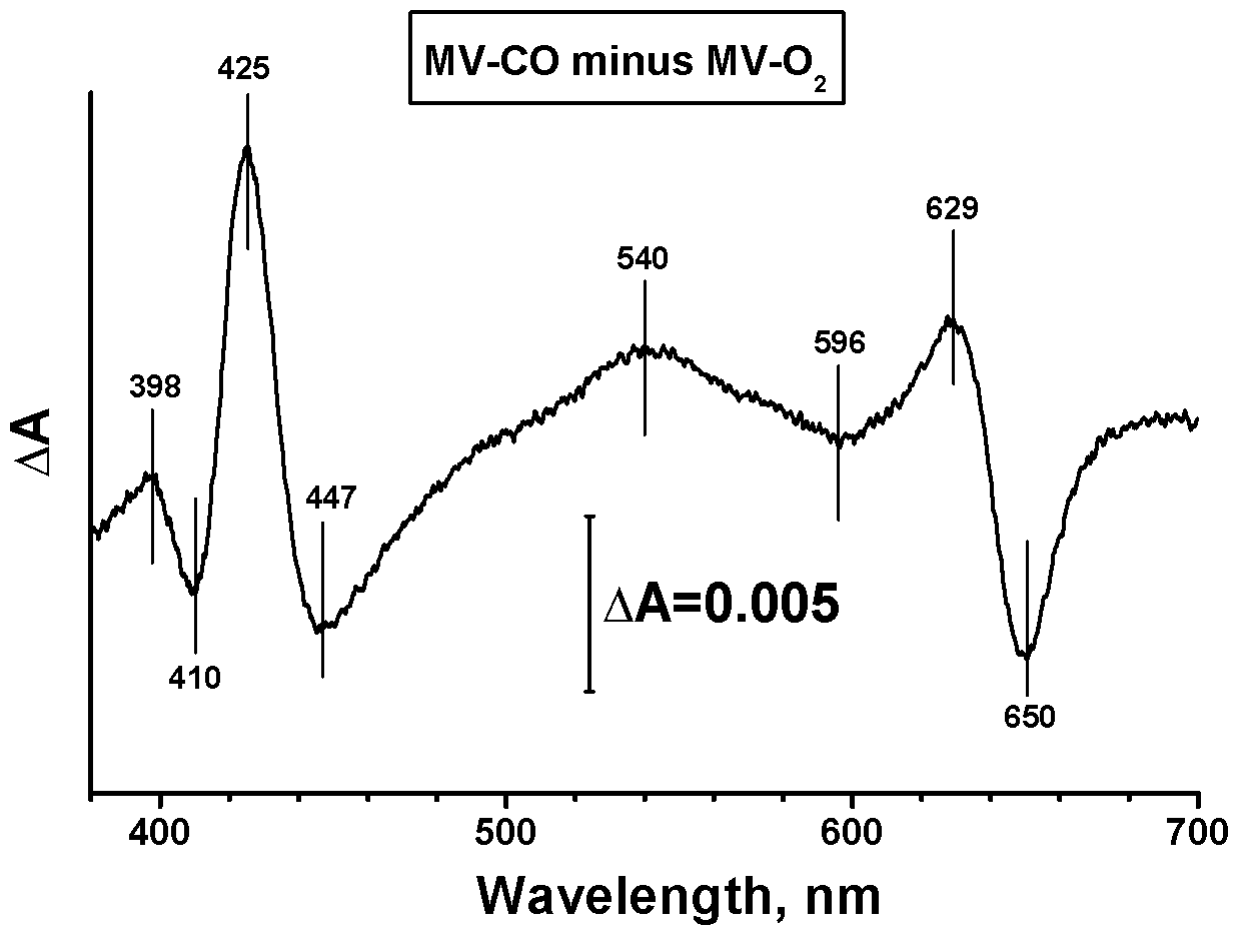
Formation of the CO compound of isolated cytochrome *bd* from *E. coli* in MV state. Shown is static difference absorbance spectrum of CO-treated enzyme *versus* a spectrum of as-isolated enzyme. The experiments were performed at 20 °C in buffer containing 50 mM Hepes, 50 mM Ches, 0.1 mM EDTA, and 0.05% sodium *N*-lauryl-sarcosinate (pH 8.0) in an optical cell of 10 mm pathway. Concentrations of enzyme and CO were 1.9 µM and 1 mM respectively.

Cytochrome *bd* is isolated from *E. coli* in the form of a stable oxy complex of ferroheme *d*, with both hemes *b* being ferric (MV-O_2_). This unique feature allows us to generate the enzyme in complex with CO (MV-CO) displacing the O_2_ molecule from heme *d*. The corresponding static difference absorbance spectrum shows a minimum at 650 nm indicative of the disappearance of the heme *d* oxy complex and maxima at 540 and 629 nm pointing to formation of the CO compound of heme *d* (Fig. 1).

### Recombination of CO with isolated cytochrome *bd* in MV state

**Figure 2:**
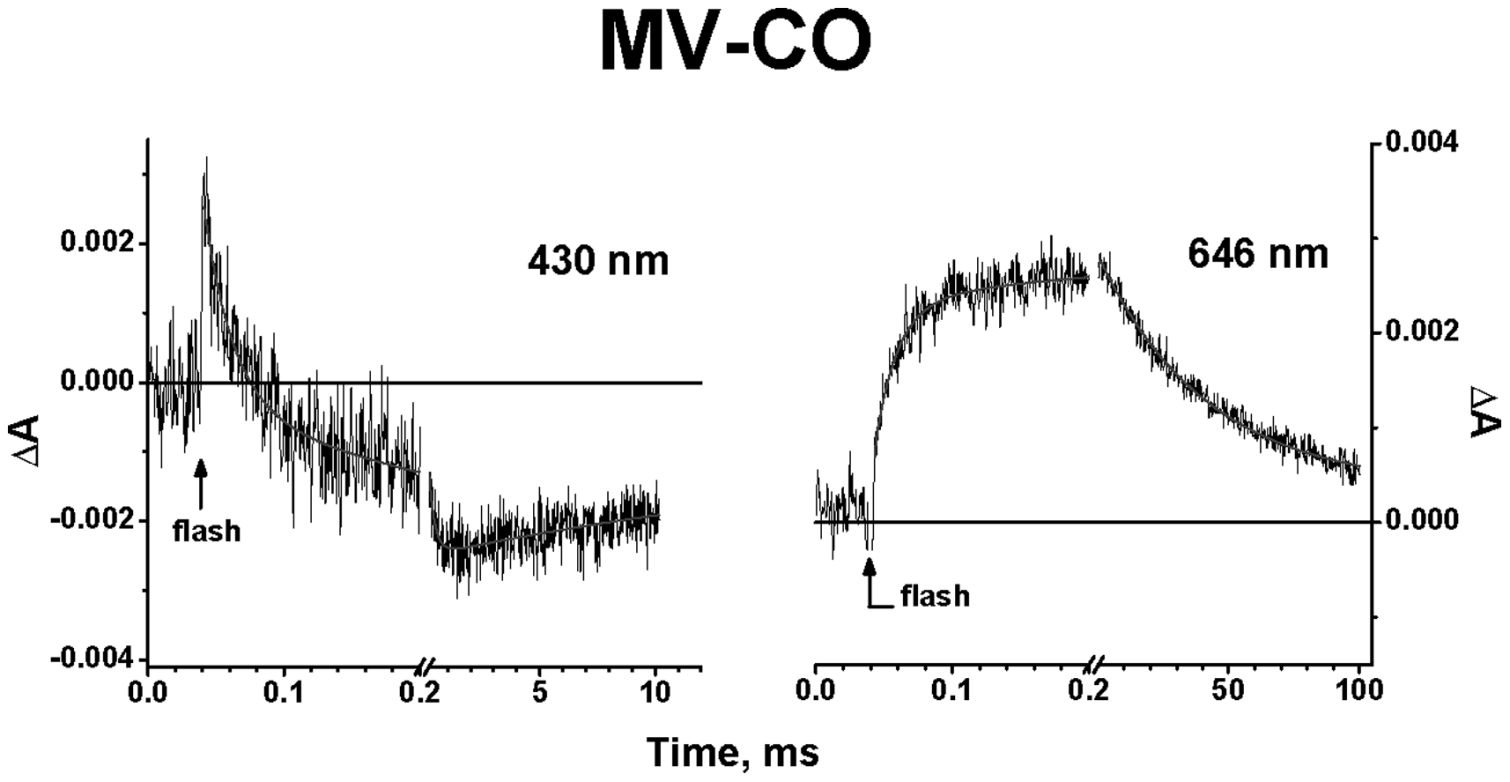
Absorbance changes accompanying photodissociation and subsequent recombination of CO with cytochrome *bd* in MV state. Kinetics at selected wavelengths. The kinetic data points (noisy traces) are shown with their best fits (smooth lines). Buffer: 50 mM Hepes, 50 mM Ches, 0.1 mM EDTA, 0.05% sodium *N*-lauryl-sarcosinate, pH 8.0. Enzyme, 1.9 µM; CO, 1 mM. Optical pathway, 10 mm; excitation, 532 nm; temperature, 20 °C.

**Figure 3:**
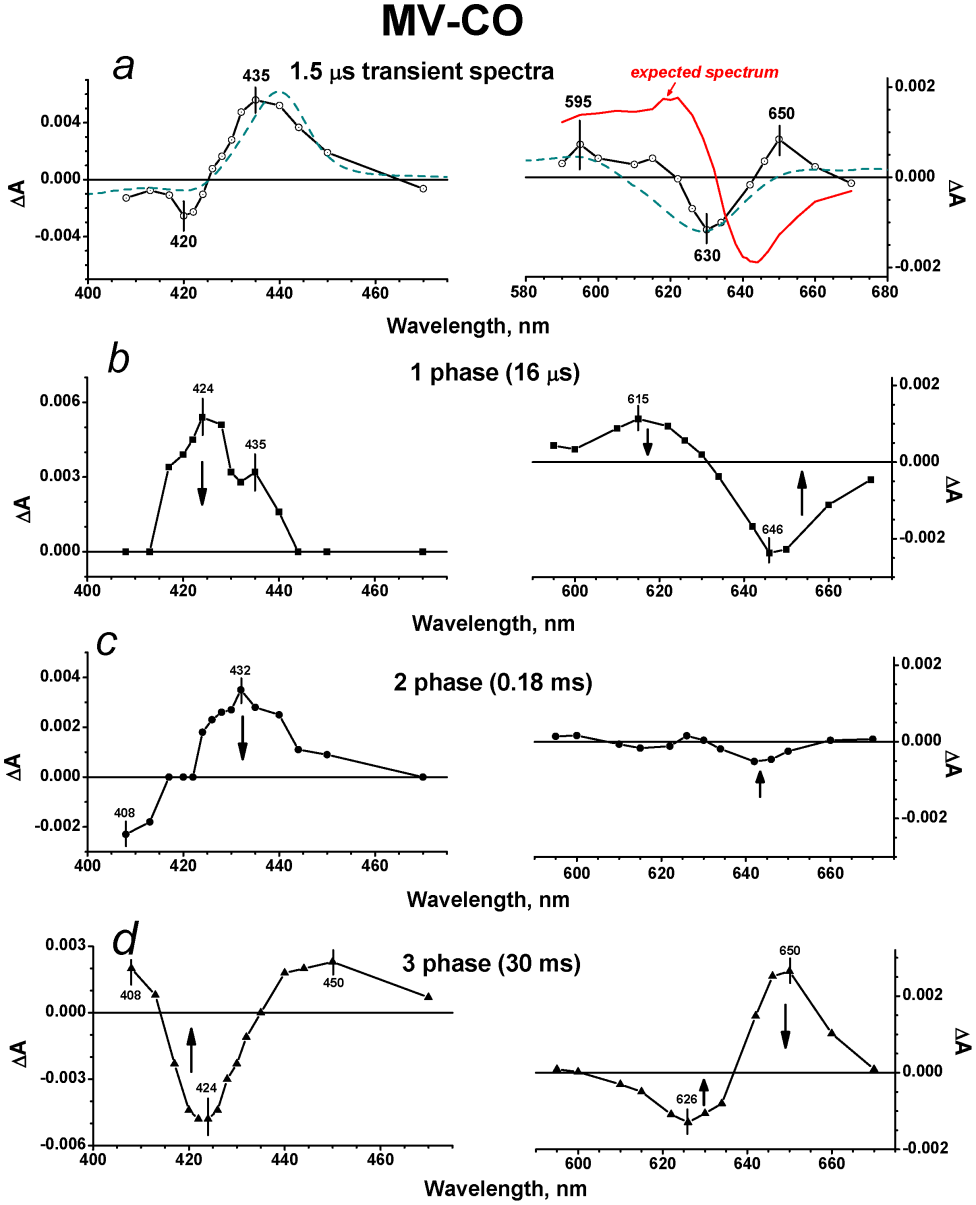
Absorbance changes following flash photolysis of the CO complex with cytochrome *bd* in MV state. (a) Transient spectra in the Soret and visible regions at a delay time of ∼1.5 µs (*versus* pre-trigger). Dark cyan dashed lines: model spectra of electron transfer from heme *d* to heme *b*_595_ constructed from individual reduced-*minus*-oxidized difference absorption spectra of the hemes published in [56]. Red solid line in *right panel*: expected spectrum. The expected spectrum is the transient spectrum for the R enzyme at a delay time of ∼1.5 µs (taken from Fig. 5a, right panel) reduced by a factor of 2.5 to account for geminate recombination of CO to heme *d* that occurs in the MV enzyme at early times [41], [43], [46]. (b), (c) and (d) Difference spectra of kinetic phases with τ ∼ 16 µs, 180 µs and 30 ms respectively. Buffer: 50 mM Hepes, 50 mM Ches, 0.1 mM EDTA, 0.05% sodium *N*-lauryl-sarcosinate, pH 8.0. Enzyme, 1.9 µM; CO, 1 mM. Optical pathway, 10 mm; excitation, 532 nm; temperature, 20 °C.

The flash-induced photodissociation and subsequent recombination of CO with the isolated cytochrome *bd* in the MV state was examined on the micro/millisecond time scale using 532-nm excitation. Upon the 15 ns laser flash, CO is immediately photodissociated from heme *d*. The kinetic traces at selected wavelengths in the visible and Soret regions are shown by Fig. 2. The immediate response to the flash is followed by the subsequent phases of the absorbance changes (Fig. 2). Global analysis of the kinetic data both in the Soret and visible regions reveals three transition steps of the absorbance changes with τ (time constant, reciprocal of rate constant, t_1/e_) ∼ 16 µs, 180 µs and 30 ms and allows us to construct spectra of kinetic components. Fig. 3 shows the spectra of phases together with the spectrum of the initial changes (1.5 µs *minus* pre-trigger).

A transient spectrum of absorbance changes at a delay time of ∼1.5 µs is shown by Fig. 3a. In the Soret band, the spectrum displays a maximum at 435 nm and a minimum at about 420 nm (Fig. 3a, left panel). In the visible region (Fig. 3a, right panel), there is a minimum at 630 nm and a maximum at about 595 nm both of which are fingerprints of heme *d* oxidation and heme *b*_595_ reduction respectively [56]. The 1.5-μs spectrum can be roughly fitted by the model spectrum of electron transfer from heme *d* to heme *b*_595_ (Fig. 3a, dark cyan dashed lines) computed using individual reduced-*minus*-oxidized difference absorbance spectra of the hemes reported in [56]. Thus it is possible that photodissociation of CO from heme *d* is followed by fast (<1.5 µs) backflow of electrons from heme *d* to heme *b*_595_, not reported earlier. If this is the case, this corresponds to the oxidation of 3–4% of heme *d* and the reduction of the same amount of heme *b*_595_ within 1.5 µs, taking into account the extinction coefficients for the difference (reduced-*minus*-oxidized) spectra of heme *b*_595_ and heme *d* [56].

The two spectra (the 1.5-μs spectrum and the model spectrum for the electron transfer) look similar but clearly are not identical (Fig. 3a). The latter may arise from the contribution of CO photolysis from a reduced heme *b* in a fraction of the MV enzyme to the 1.5-μs spectrum. Besides, one should be taken into account that transient spectra may not be fully identical to the corresponding equilibrium spectra of the same processes. Note that the model spectrum of electron transfer given here was generated from the equilibrium spectroelectrochemical titration data [56]. Finally, the proposed ligand interchange in the coordination sphere of heme *d* (binding of unknown L simultaneously with the CO photodissociation, see the next paragraph) may add some minor change in absorbance to the 1.5-μs spectrum.

It is noteworthy that a photolysis spectrum of absorbance changes accompanying a ligation state change in heme *d* in response to dissociation of CO is not seen in the visible region. (In this region the absorbance changes induced by CO dissociation from heme *d* are well known and differ spectrally from those caused by the oxidation of heme *d* [10].) There is almost no initial jump in the kinetic trace at 646 nm (Fig. 2, right panel) that is close to the maximum in the difference spectrum of CO binding to heme *d* [35]. Indeed, upon CO binding to heme *d* in the R enzyme from *E. coli* or *Azotobacter vinelandii*, the static difference absorbance spectrum with a maximum at 642–644 nm and a minimum at 622–624 nm in the visible region is persistently observed [35], [42], [57], [58]. The corresponding inverted transient spectrum should be anticipated upon photodissociation of CO from heme *d* (Fig. 3a, right panel, red solid line). Even if the picosecond and nanosecond phases of geminate recombination of CO to heme *d* reported earlier with cytochrome *bd* in the MV state [41], [43], [46] are taken into account, the absorbance changes in the visible region due to photolysis of CO from the MV enzyme, resolved at 1–2 µs after the flash, should have been inverted and at least two times higher in magnitude compared to what is actually observed (Fig. 3a, right panel). Thus, the apparent contribution of the absorbance changes associated with CO dissociation from the distal side of heme *d* to the 1.5-μs spectrum is unexpectedly small if any (Fig. 3a, right panel). We propose that these absorbance changes are minimized due to the flash-induced binding of a ligand (L) other than CO to the proximal side of heme *d* resulting in transient formation of the pentacoordinate complex, Fe_d_^2+^-L (see Discussion). These two effects (dissociation of CO from and binding of L to heme *d*) may balance out.

Although we suggest that the spectra of binding of L and CO to heme *d* are very similar thus masking the general shape of absorbance changes and resembling each other, it is possible that they are not fully identical. The latter may be one more reason why the observed 1.5-μs spectrum and the model spectrum for the electron transfer are similar but do not coincide (Fig. 3a).

The kinetic spectrum of the 16-μs rapid phase shows a minimum at 646 nm together with a maximum at about 615 nm in the visible region (Fig. 3b, right panel). This spectral shift in the visible is similar to the spectral changes induced by CO binding to heme *d* [35], [42], [43], [57], [58] and therefore may be generally attributed to recombination of CO with heme *d*. The magnitude of the absorption changes associated with the observed recombination is virtually the same as that expected from the difference spectra of CO binding to heme *d* taking into account geminate recombination of CO to heme *d* (∼50–70%) occurring at early times [41], [43], [46]. In the Soret band, there are two maxima, at 435 and 424 nm (Fig. 3b, left panel). This spectrum is very similar to the previously reported spectrum of the 14-μs component (see Fig. 2C in [46]). This was shown to comprise bimolecular recombination of CO with heme *d* and the electron transfer (backflow) from heme *d* to heme(s) *b* in a small fraction of the hemoprotein [46]. In agreement with that analysis [46], we also conclude that the overall absorbance decay in the Soret region observed here (Fig. 3b, left panel) reflects recombination of CO with heme *d* and the electron backflow from heme *d* to heme(s) *b* in a minor fraction of the cytochrome *bd* population.

The intermediate phase with τ ∼ 180 µs shows the Soret spectrum with a broad maximum at about 432 nm and a minimum around 408 nm (Fig. 3c, left panel). This spectrum is consistent with the spectrum of the 280-μs component (140–290 µs in different experiments), observed before in [46]. The transition can be assigned to re-reduction of heme *d* by the electron returning from a *b*-type heme and concurrent recombination of CO with heme *d* in a fraction of the enzyme molecules, in agreement with [46]. The changes in the visible showing a minimum at 642 nm and a smaller maximum at 626 nm (Fig. 3c, right panel) are in line with such assignment.

The spectrum of the slow phase with τ ∼ 30 ms (21–41 ms in different experiments) shows a minimum at approximately 424 nm in the Soret region (Fig. 3d, left panel). This spectrum seems to resemble a CO-bound reduced-*minus*-reduced spectrum of a *b*-type heme [59]. In the visible region, there is a minimum at about 626 nm and a maximum near 650 nm (Fig. 3d, right panel). The line shape and magnitude of the spectrum of the 30-ms phase in the visible region (Fig. 3d, right panel) are similar to those of the spectrum of the 16-μs phase that generally reflects recombination of CO to heme *d* (Fig. 3b, right panel), but the direction of the signal development is opposite. Thus the overall absorbance changes of the 30-ms phase may be attributed to (i) dissociation of L from the proximal side of heme *d* yielding the pentacoordinate heme *d*-CO adduct (see Discussion), and (ii) recombination of CO with a reduced heme *b* in a minor fraction of the enzyme in the MV state.

### Recombination of CO with isolated cytochrome *bd* in R state

Photodissociation and further recombination of CO with cytochrome *bd* in the R state were studied under the same conditions as for the MV enzyme. The R state of the enzyme obtained by addition of dithionite to the air-oxidized cytochrome *bd* is characterized by a typical difference absorbance spectrum (reduced *minus* “air-oxidized”) (not shown, but see [12], [57], [58]).

**Figure 4:**
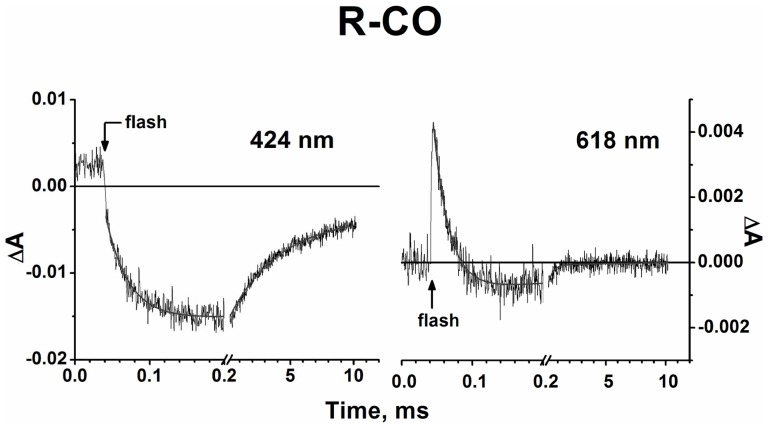
Absorbance changes accompanying photodissociation and subsequent recombination of CO with cytochrome *bd* in R state. Kinetics at selected wavelengths. The kinetic data points (noisy traces) are shown with their best fits (smooth lines). Buffer: 50 mM Hepes, 50 mM Ches, 0.1 mM EDTA, 0.05% sodium *N*-lauryl-sarcosinate, pH 8.0. Enzyme, 1.9 µM; CO, 1 mM. Optical pathway, 10 mm; excitation, 532 nm; temperature, 20 °C.

**Figure 5:**
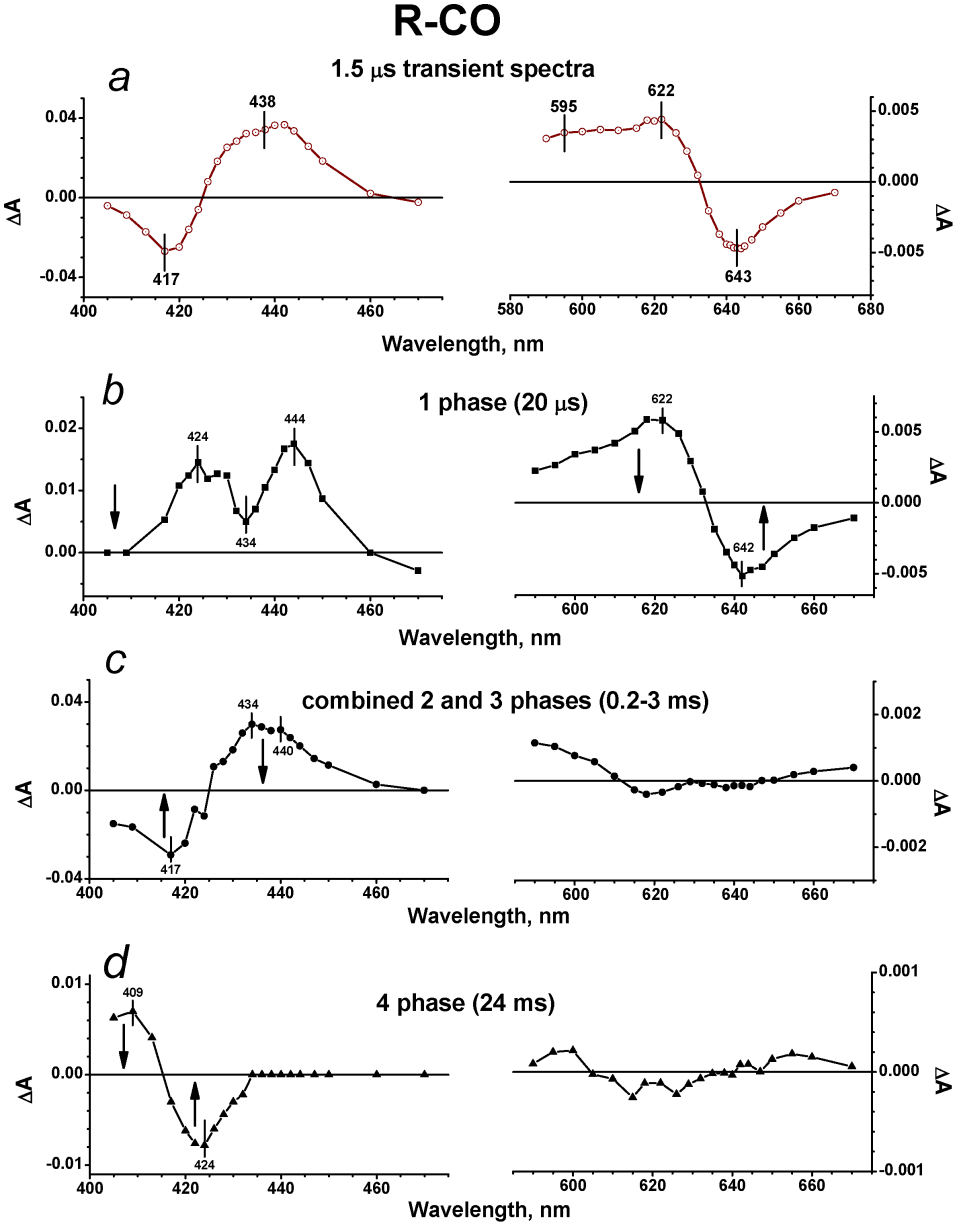
Absorbance changes following flash photolysis of the CO complex with cytochrome *bd* in R state. (a) Transient spectra in the Soret and visible regions at a delay time of ∼1.5 µs (*versus* pre-trigger). (b), (c) and (d) – Difference spectra of 20 µs, combined 0.2–3 ms and 24 ms kinetic phases respectively. Buffer: 50 mM Hepes, 50 mM Ches, 0.1 mM EDTA, 0.05% sodium *N*-lauryl-sarcosinate, pH 8.0. Enzyme, 1.9 µM; CO, 1 mM. Optical pathway, 10 mm; excitation, 532 nm; temperature, 20 °C.

Fig. 4 shows the kinetic traces at selected wavelengths in the visible and Soret regions, which refer to cytochrome *bd* in the R-CO state, subjected to a 532-nm laser flash. Global analysis of the kinetic data both in the Soret and visible regions (Fig. 4) reveals transition steps of the post-flash absorbance changes with τ ∼ 20 µs, 0.2-3 ms and 20-40 ms and allows us to construct spectra of kinetic components (Fig. 5). Although the time characteristics of the transition processes upon the measurements with the two initial states (MV and R) of the enzyme are apparently similar, further analysis of the spectra suggests that the nature of the processes is different. Unlike cytochrome *bd* in the MV state, in the R enzyme CO can bind not only to heme *d* but also to part of hemes *b.* The latter is the cause of the additional phases of recombination in case of cytochrome *bd* in the R state.

Fig. 5a (left panel) shows a transient spectrum in the Soret at a delay time of ∼1.5 µs. The spectrum displays a broad maximum centered around 438 nm and a minimum at 417 nm. This suggests CO dissociation from heme *d* and part of hemes *b*. The concomitant transient spectrum in the visible displays a minimum at 643 nm and a maximum at 622 nm (Fig. 5a, right panel) that is a typical difference spectrum (inverted) induced by binding of CO to heme *d* [35]. In addition, there is a shoulder around 595 nm pointing to formation of unliganded ferrous heme *b*_595_ after CO dissociation [42] in ∼5% of the enzyme fraction (4–7% in different experiments). Accordingly, there is an unresolved but clear initial jump in the kinetic trace at 618 nm (Fig. 4, right panel) characteristic of CO photodissociation from heme *d*, which is nearly absent in the case of the MV enzyme (Fig. 2, right panel).

A “W-shaped” spectrum of the rapid phase in the Soret as well as a typical shift of the heme *d* α–band in the visible (Fig. 5b) are the characteristics of CO binding to heme *d* in the R enzyme [35], [44]. Therefore, this spectrum can be undoubtedly assigned to CO binding to heme *d* in the R cytochrome *bd*. The characteristic time of this phase (τ∼20 µs at 1 mM CO) corresponds to a second-order rate constant of CO recombination to heme *d* of ∼ 5×10^7^ M^−1^·s^−1^, in agreement with the previously reported values for cytochrome *bd* from *E. coli* [60], [61] and *A. vinelandii* [42], [62]. As shown earlier [41], [43], [46], unlike the MV state, the R state reveals no geminate but only bimolecular recombination after photodissociation of CO from heme *d*. Therefore, the spectrum of the 20-μs phase should be at least two times higher in magnitude than its counterpart in the MV enzyme.

The intermediate part of recombination consists of the two phases with similar spectra with τ in the range of 0.2–3 ms. The spectrum of the combined phases shows a minimum at about 417 nm and a maximum near 434 nm in the Soret and some minor changes in the visible (Fig. 5c). The changes in the Soret band are typical of CO binding to a *b*-type heme [59]. Thus, it seems reasonable to assign this spectrum as arising from CO recombination with heme *b*. The magnitude of the absorbance changes corresponds to CO rebinding with 15–20% of a *b*-type heme provided that the extinction coefficient for a difference spectrum (γ-peak *minus* γ-trough) induced by CO binding to a reduced *b*-type hemoprotein is ∼ 150–200 mM^−1^·cm^−1^ [59]. We suggest that this is mostly heme *b*_558_ in view of the fact that under similar conditions addition of 1 mM CO to cytochrome *bd* in the R state induces a 15% decrease in the MCD signal of heme *b*_558_ [35]. As shown earlier, its sixth axial ligand Met_393_ bonds a polypeptide to the iron atom of the heme rather poorly and can be displaced by a stronger ligand like CO in part of the enzyme molecules [44].

The spectrum of the slow phase with τ ∼ 24 ms (18–30 ms in different experiments) shows a minimum at 424 nm and a maximum at 409 nm in the Soret and some minor changes in the visible (Fig. 5d). The changes in the Soret can be reasonably fitted by migration of CO from heme *d* to heme *b*_595_ in ∼5% of cytochrome *bd* population using the spectra of CO interactions to heme *d* [43] and heme *b*_595_ [42] (not shown). The concomitant changes in the visible are small but reproducible. They show a maximum around 595 nm (conversion of unliganded heme *b*_595_ into its CO compound), a minimum around 624 nm and a maximum at about 642 nm (dissociation of CO from heme *d*). These visible spectral features support the interpretation of the changes in the Soret band.

## Discussion

We compared photodissociation and subsequent rebinding of CO with the isolated cytochrome *bd* from *E. coli* in the MV and R states probing both the Soret band and the visible region. The measurements in the visible region allow better understanding of the changes in the Soret band. Using 532-nm excitation, the absorption bands of all three hemes (*b*_558_, *b*_595_, *d*) should be excited [56]. The data are consistent with previously published experiments performed with nanosecond time resolution but limited to the Soret band [46]. In both redox states of cytochrome *bd*, CO recombines to heme *d* with τ  =  16–20 µs (at 1 mM CO). In addition, in a small fraction of the MV enzyme, this process is accompanied by the electron backflow from heme *d* to a *b*-type heme and the reversal of that transfer with τ of 16 µs and 180 µs respectively (Fig. 3b,c). The same events occurred with very similar time constants were observed in previous study [46]. The current study, however, revealed additional phases which were not observed in the previous work [46]. The reason for this apparent discrepancy is the differences in the experimental setup. The work of Rappaport et al. [46] utilized excitation at 640 nm, near the α band of heme *d*, that allowed photolysis of the CO compound of heme *d* only. On the contrary, the present experiments were performed under non-selective excitation conditions at which the CO from other ferrous hemes, such as *b*_558_ and *b*_595_, can be ‘flashed’ off. Furthermore, the protein environment was different in the two studies, i.e. detergent molecules (this work) *versus* natural lipids [46]. The membrane environment was shown [44] to affect significantly the CO-binding properties of a *b*-type heme in cytochrome *bd*.

Thus, in the R enzyme, following photolysis at 532-nm excitation, CO first recombines to heme *d* (20 µs) and then to heme *b*_558_ (with τ in the range of 0.2–3 ms) in a fraction of the enzyme in which the Fe_b558_–Met bond is weakened and/or broken. The conclusion that CO at a high concentration can react with ferrous heme *b*_558_ is consistent with the MCD [35] and CO titration [44] studies. In the slowest recombination phase (24 ms) CO migrates from heme *d* to heme *b*_595_ in ∼5% of the enzyme population, in agreement with recent work [48]. These data support a model of the cytochrome *bd* active site in which hemes *b*_595_ and *d* form the common oxygen reducing pocket but with the negative cooperativity of ligand binding, heme *d* having a higher affinity. If some heme *b*-CO were present in the sample used in [46], it could not be photolyzed at 640-nm excitation.

In case of the MV-CO enzyme, the line shape of the transient spectrum at a delay time of 1.5 µs (Fig. 3a) implies that the flash-induced CO dissociation from heme *d* is followed by fast (within 1.5 µs) electron transfer from heme *d* to heme *b*_595_ that precedes the electron flow to heme *b*_558_. Indeed, this transient spectrum can be roughly approximated by the model spectrum of electron transfer from heme *d* to heme *b*_595_ (Fig. 3a) in 3–4% of the enzyme. This phase was not reported in [46]. As noted above, the difference can be explained by different experimental conditions used in this work and in [46]. It is worth noting that the very fast (in the nanosecond regime) electron transfer between the closely lying hemes *o*_3_ and *b* in the *E. coli* cytochrome *bo*_3_ [63] and hemes *a*_3_ and *a* in mammalian cytochrome *c* oxidase [64] has been recently reported. Experiments with higher time resolution under varying conditions are needed to further validate existence of the fast electron transfer in the MV cytochrome *bd* and determine its time constant.

**Figure 6:**
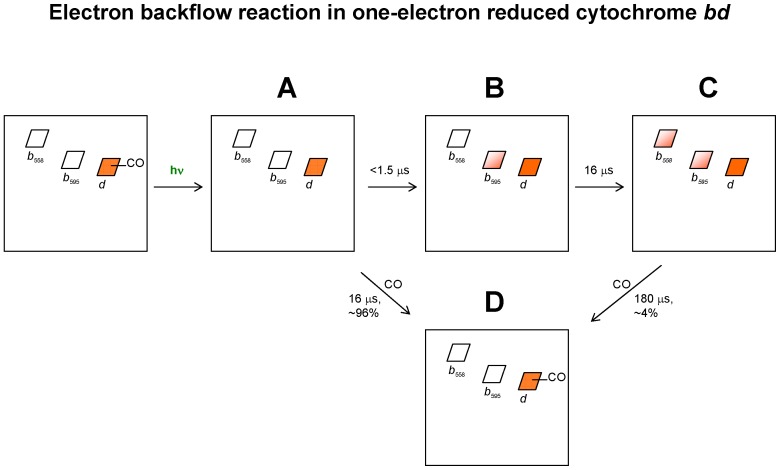
Proposed scheme for backflow of electrons in cytochrome *bd* in MV state. Each rhombus represents one of the three hemes in cytochrome *bd*. Filled or semi-filled rhombus denotes that site is in the reduced state. Empty rhombus denotes that site is in the oxidized state. During the backflow reaction (observed in ∼4% of the enzyme at 1 mM CO) the electron from heme *d* transfers sequentially to heme *b*_595_ (A→B transition, τ<1.5 µs) and heme *b*_558_ (B→C transition, τ∼16 µs). Finally, the electron equilibrates between the redox sites with respect to their redox potentials. A→D and C→D transitions describe recombination of CO to 96% and 4% of heme *d*, respectively.

Fig. 6 summarizes electron transfer reactions induced by photolysis of the CO compound of the MV cytochrome *bd* at 1 mM CO. Following the proposed fast electron transfer from heme *d* to heme *b*_595_ with τ<1.5 µs (Fig. 6, A→B transition), the slower back electron transfer with τ∼16 µs occurs. That transfer results in the oxidation of heme *d* and partial reduction of both hemes *b* (Fig. 6, B→C transition) in a small fraction of the enzyme. The reversal of electron backflow occurs with τ ∼ 180 µs (Fig. 6, C→D transition).

**Figure 7:**
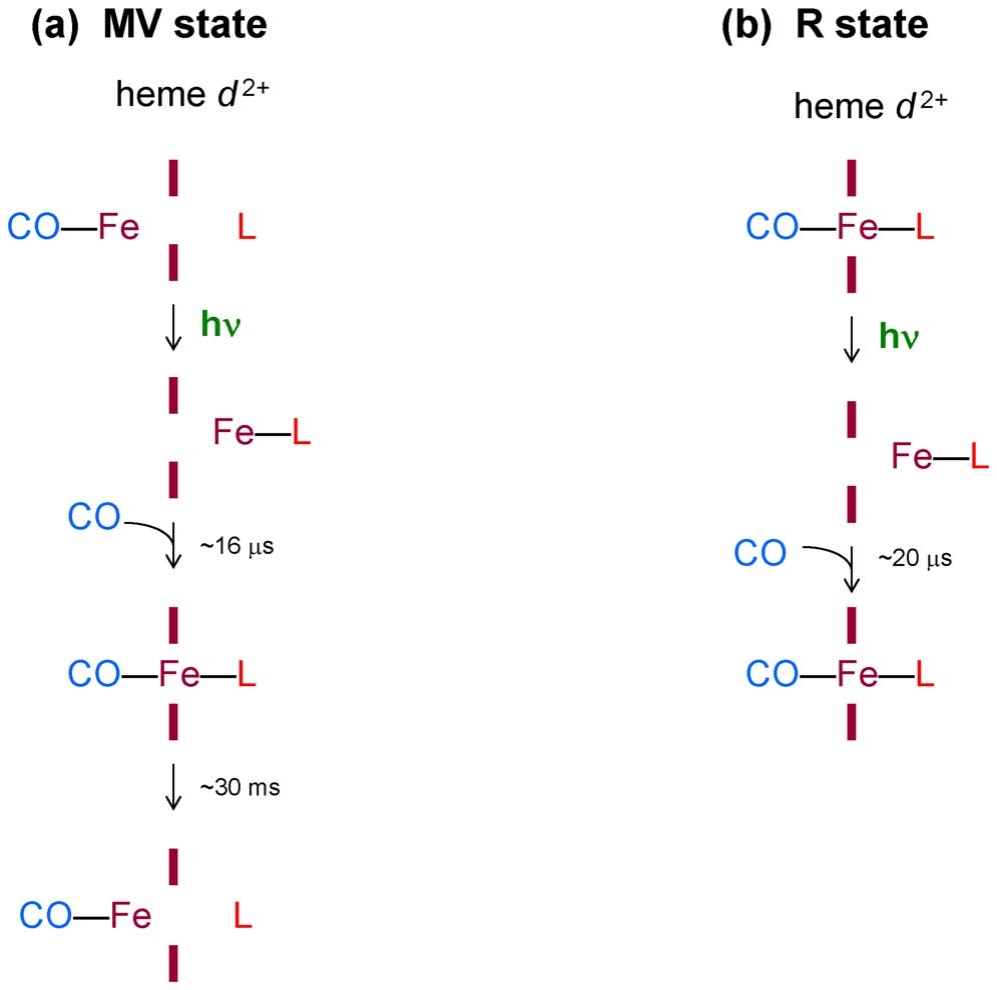
Proposed intermediate states of the *E. coli* cytochrome *bd* during photolysis of CO and its subsequent recombination. The minimal scheme shows the porphyrin plane of heme *d*, the central iron atom, changes in heme ligation and the time constants for the resolved transitions of heme *d*. MV (a) and R (b) are the redox states of the enzyme. In the MV state of the enzyme, photodissociation of CO from heme *d* is accompanied by binding of L at the opposite side of the heme. CO recombines with heme *d* with τ∼16 µs yielding a transient hexacoordinate state (CO-Fe^2+^-L). Then L is dissociated from heme *d* with τ∼30 ms. In the R state of the enzyme, L is a permanent undissociable proximal ligand to heme *d*. In this case, there are two transitions with regard to heme *d*: after photolysis CO leaves the heme pocket and then returns to heme *d* with τ∼20 µs.

Remarkably, the spectrum of photolysis of CO from the MV enzyme (the 1.5 µs-transient, Fig. 3a), is not sufficient to fit as the sum of two processes, dissociation of CO from heme *d* and electron backflow from heme *d* to heme *b*_595_. The changes in the visible region show nearly pure electron transfer reaction, whereas the photodissociation of CO from heme *d* is hardly seen. To explain this fact, we propose that, in the MV enzyme, CO dissociation from the distal side of heme *d* is accompanied by simultaneous binding of an internal ligand (L) to the opposite, proximal side of heme *d* producing the transient pentacoordinate Fe_d_–L species (Fig. 7a), and the absorbance changes induced by these two events may mostly cancel out. Then in the 16-μs phase CO returns to heme *d* yielding the transient hexacoordinate CO–Fe_d_–L species (Fig. 7a) that is manifested as the kinetic spectrum with a minimum at ∼646 nm and a maximum at ∼616 nm in the visible together with the overall maximum in the Soret band (Fig. 3b). Finally, in the 30-ms phase L is detached from heme *d* and the system is returned to its initial state before photolysis, CO–Fe_d_ (Fig. 7a).

The 30-ms phase was not reported in [46] because of the differences in the experimental setup. As noted by one of the reviewers, the spectrum of the 30-ms phase in the Soret region resembles a CO-bound reduced-*minus*-reduced spectrum of a *b*-type heme [59] and could thus be due to some *b*-CO present in the MV-CO sample. The unselective 532-nm excitation would dissociate this bond, as we observed for the R-CO state, but heme *d*-selective excitation (640 nm) would not. However, it should be stressed that this event makes but minor contribution to the observed spectrum of the 30-ms phase in the visible region. The latter spectrum (Fig. 3d, right panel) is similar to the (inverted) spectrum of the 16-μs phase (Fig. 3b, right panel). The visible region was not examined in [46]. Thus we can conclude that the 30-ms phase comprises (i) dissociation of L from the proximal side of heme *d* yielding the pentacoordinate heme *d*-CO adduct, and (ii) recombination of CO with a ferrous heme *b* in a small fraction of the MV sample.

The question arises what is the nature of L? Two simplest possibilities may be considered:

(1) L is O_2_. Indeed, the spectrum of the slow, 30-ms phase (Fig. 3d) is apparently similar to the static spectrum that shows the displacement of O_2_ from the heme *d* site by CO (Fig. 1). One may propose the existence of an intraprotein cavity connected to heme *d* that can serve as a reservoir for O_2_ that is not removed upon purging the sample with argon and CO. This would explain why O_2_ does not leave the protein with each flash but returns to the cavity. Such a cavity might play a role in protein function, since cytochrome *bd* is a high-O_2_-affinity oxidase [15], [16] that enables bacteria to survive in a microaerobic environment [10]. Since so far it is difficult to determine with certainty that O_2_ could not be removed, or is disconnected from the ‘outside’ world, this suggestion is not very likely, although cannot be discarded.

(2) L is an endogenous protein ligand. This would be consistent with an earlier report [65] that spectral changes accompanying exchange or binding of an endogenous ligand to heme *d* upon reduction of cytochrome *bd* resemble those induced by a diatomic gas like CO, O_2_ or NO. This hypothesis is also in agreement with the interpretation of a fifth ligand dissociating from heme *d* upon O_2_ binding suggested previously [36].

If the second possibility is correct, it would be reasonable to expect the same behavior of L (binding to/detachment from heme *d*) for the R enzyme as well. However, this is not the case. The spectra of the slowest phases of recombination in the visible region are clearly different (cf. right panels in Fig. 3d and 5d) and therefore cannot reflect the same reaction, dissociation of L from heme *d* (Fig. 7). As stated above, the spectral changes due to CO recombination with heme *d* in the MV and R states during the 16–20 µs phase are very similar. Hence, they may suggest the same (or very similar) change in the ligation state of heme *d*. Meanwhile, the direction of the signal development in the 20-μs phase of recombination of CO to heme *d* in the R state in the visible region is inverted with regard to the 1.5-μs photolysis spectrum (Fig. 5a,b, right panels). According to the proposed model, this is only possible if L is a permanent undissociable proximal ligand to heme *d* in the R state of the enzyme, while it is a transient heme *d* iron ligand in the MV state of the enzyme (Fig. 7). It is possible that the redox state of heme *b*_595_ determines the ligation of heme *d* iron. Since the three dimensional structure of cytochrome *bd* is not available and the axial ligand of heme *d* is not identified yet, it is difficult to define at present the chemical nature of L. This intrinsic ligand is unlikely to be His as the iron to His bond in the CO complex is strong. A residue that plays this role might be a highly conserved Glu_99_ in subunit I provided this is the axial ligand to the heme *d* iron as proposed in [34]. Further work is needed to identify the exact nature of L.
